# Knockdown of Asparagine Synthetase A Renders *Trypanosoma brucei* Auxotrophic to Asparagine

**DOI:** 10.1371/journal.pntd.0002578

**Published:** 2013-12-05

**Authors:** Inês Loureiro, Joana Faria, Christine Clayton, Sandra Macedo Ribeiro, Nilanjan Roy, Nuno Santarém, Joana Tavares, Anabela Cordeiro-da-Silva

**Affiliations:** 1 Parasite Disease Group, Instituto de Biologia Molecular e Celular da Universidade do Porto, Porto, Portugal; 2 Zentrum für Molekulare Biologie der Universität Heidelberg, DKFZ-ZMBH Alliance, Heidelberg, Germany; 3 Protein Crystallography Group, Instituto de Biologia Molecular e Celular da Universidade do Porto, Porto, Portugal; 4 Ashok and Rita Patel Institute of Integrated Study and Research in Biotechnology and Allied Sciences, New Vallabh Vidyanagar, Gujarat, India; 5 Departamento de Ciências Biológicas, Faculdade de Farmácia da Universidade do Porto, Porto, Portugal; Institut Pasteur de Montevideo, Uruguay

## Abstract

Asparagine synthetase (AS) catalyzes the ATP-dependent conversion of aspartate into asparagine using ammonia or glutamine as nitrogen source. There are two distinct types of AS, asparagine synthetase A (AS-A), known as strictly ammonia-dependent, and asparagine synthetase B (AS-B), which can use either ammonia or glutamine. The absence of AS-A in humans, and its presence in trypanosomes, suggested AS-A as a potential drug target that deserved further investigation. We report the presence of functional AS-A in *Trypanosoma cruzi* (*Tc*AS-A) and *Trypanosoma brucei* (*Tb*AS-A): the purified enzymes convert L-aspartate into L-asparagine in the presence of ATP, ammonia and Mg^2+^. *Tc*AS-A and *Tb*AS-A use preferentially ammonia as a nitrogen donor, but surprisingly, can also use glutamine, a characteristic so far never described for any AS-A. *Tb*AS-A knockdown by RNAi didn't affect *in vitro* growth of bloodstream forms of the parasite. However, growth was significantly impaired when *Tb*AS-A knockdown parasites were cultured in medium with reduced levels of asparagine. As expected, mice infections with induced and non-induced *T. brucei* RNAi clones were similar to those from wild-type parasites. However, when induced *T. brucei* RNAi clones were injected in mice undergoing asparaginase treatment, which depletes blood asparagine, the mice exhibited lower parasitemia and a prolonged survival in comparison to similarly-treated mice infected with control parasites. Our results show that *Tb*AS-A can be important under *in vivo* conditions when asparagine is limiting, but is unlikely to be suitable as a drug target.

## Author Summary

The amino acid asparagine is important not only for protein biosynthesis, but also for nitrogen homeostasis. Asparagine synthetase catalyzes the synthesis of this amino acid. There are two forms of asparagine synthetase, A and B. The presence of type A in trypanosomes, and its absence in humans, makes this protein a potential drug target. Trypanosomes are responsible for serious parasitic diseases that rely on limited drug therapeutic options for control. In our study we present a functional characterization of trypanosomes asparagine synthetase A. We describe that *Trypanosoma brucei* and *Trypanosoma cruzi* type A enzymes are able to use either ammonia or glutamine as a nitrogen donor, within the conversion of aspartate into asparagine. Furthermore, we show that asparagine synthetase A knockdown renders *Trypanosoma brucei* auxotrophic to asparagine. Overall, this study demonstrates that interfering with asparagine metabolism represents a way to control parasite growth and infectivity.

## Introduction

Asparagine is a naturally occurring non-essential amino acid found in many proteins. Due to its high nitrogen/carbon ratio, asparagine is likely to be linked to nitrogen homeostasis and protein biosynthesis [1]. AS is the protein involved in asparagine biosynthesis. There are two distinct types of AS, AS-A and AS-B, encoded by *asnA* and *asnB* genes, respectively. AS-A encoding genes have been reported in archaea [2], [3], prokaryotes [4]–[7], and in the protozoan parasite *Leishmania* [8]. The AS-B encoding gene is present in prokaryotes [5], [9] and also in eukaryotes, including mammalian cells [10], [11], yeasts [12], algae [13], and higher plants [14]. Both types catalyze the ATP-dependent conversion of aspartate into asparagine. While AS-B can use both ammonia and glutamine (reaction B) as amide nitrogen donors [5], [15]–[20], *Escherichia coli* (*E. coli*) AS-A was reported to be dependent strictly on ammonia (reaction A) [21], [22].

- ATP + L-aspartate + NH4^+^  = > AMP + diphosphate + L-asparagine
- ATP + L-aspartate + L-glutamine [or NH4^+^]  = > AMP + diphosphate + L-asparagine + L-glutamate

AS-A and AS-B share no sequence or structural similarities. Their three-dimensional structures provided important information concerning their distinct catalytic mechanisms [2], [23]–[25]. AS-A exists as a dimer where each monomer has a core of eight β-strands flanked by α-helices, resembling the catalytic domain of class II aminoacyl-tRNA synthetases such as aspartyl-tRNA synthetase [24]. AS-A synthesizes asparagine in two steps: the β-carboxylate group of aspartate is first activated by ATP to form an aminoacyl-AMP, followed by amidation by a nucleophilic attack with an ammonium ion [2]. The AS-B enzyme also forms a dimer, but each monomer contains two distinct domains, each of which contains a catalytic site. The N-terminal site catalyzes the conversion of glutamine into glutamic acid and ammonia, while aspartate reacts with ATP in the C-terminal site, generating the intermediate β-aspartyl-AMP [26], [27]. Similarly to other glutamine dependent amidotransferases, ammonia released in the N-terminal domain of the enzyme travels through an intramolecular tunnel connecting the active sites, and reacts with the reactive acyladenylate intermediate to produce asparagine [28].

An open reading frame encoding a putative AS-A is present in the genome of the protozoan parasites, *Trypanosoma cruzi* (*T. cruzi*) and *Trypanosoma brucei* (*T. brucei*) [29]–[31]. *T. cruzi* and *T. brucei* are transmitted to a mammalian host through an invertebrate vector, and are responsible for Chagas disease and African sleeping sickness, respectively. Disease control is dependent on drug therapy, but treatment options are limited, both by high toxicity and recent emergence of drug resistance [32]–[34]. Vaccines for *T. brucei* infections are unlikely to be developed not only because of extensive antigenic variation [35], but also because infections compromise host humoral immune competence [36].

Trypanosome AS-A might be a drug target due to the absence of a homologue in humans [8]. AS-A is important in other microorganisms. For example, *asnA* is an essential gene in *Haemophilus influenzae* (DEG10050178) [37], and is strongly up-regulated in *Pasteurella multocida* during host infection [38], and when *Klebsiella aerogenes* is grown in an amino acid-limited but ammonia rich environment [5]. We therefore undertook biochemical and genetic studies of AS-A in trypanosomes to ascertain its biological role and evaluate its potentiality as drug target.

## Materials and Methods

### Ethics statement

All experiments involving animals were carried out in accordance with the IBMC.INEB Animal Ethics Committees and the Portuguese National Authorities for Animal Health guidelines, according to the statements on the directive 2010/63/EU of the European Parliament and of the Council. IL, JT and ACS have an accreditation for animal research given by the Portuguese Veterinary Direction (Ministerial Directive 1005/92).

### Parasite culture

Procyclic and bloodstream forms of *Trypanosoma brucei brucei* Lister 427 were used. Procyclic forms were grown in MEM-Pros medium supplemented with 7.5 µg/ml hemin, 10% fetal calf serum (FCS) and 100 IU/mL of penicillin/streptomycin at 27°C, with cell densities between 5×10^5^ cells/ml to 1–2×10^7^ cells/ml. Bloodstream forms were grown in complete HMI-9 medium (supplemented with 10% FCS and 100 IU/mL of penicillin/streptomycin) [39] in vented tissue culture flasks; these cultures were diluted when cultures reached the cell density of 2×10^6^/ml and incubated in a humidified atmosphere of 5% CO2, at 37°C. Bloodstream RNAi cell cultures were supplemented with 7.5 µg/ml hygromycin and 0.2 µg/ml phleomycin.

### Cloning of *T. brucei* and *T. cruzi ASA* genes

*T. brucei* asparagine synthetase A (*TbASA*) and *T. cruzi* asparagine synthetase (*TcASA*) genes were obtained by performing PCR on genomic DNA from *Trypanosoma brucei brucei* TREU927 and *Trypanosoma cruzi* CL Brener Non-Esmeraldo-like. Fragments of the open reading frames of *TbASA* (Tb927.7.1110; chromosome Tb927_07_v4; 28861 to 289067) and *TcASA* (Tc00.1047053503625.10; chromosome TcChr29-P; 687159–688206) were PCR-amplified using a Taq DNA polymerase with proofreading activity (Roche). The sequences of the primers were as follows: sense primer 5′ - CTAATTACATATGGGCGACGACGGTTATTC - 3′ and antisense primer 5′ - CCCAAGCGAATTCTTACAACAAATTGTGC - 3′, sense primer 5′ - CAAT TTGCATATGACATCGGGAGATCC - 3′ and antisense primer 5′ - CCCAAGCAAGCTTTCACAGCAAGGG - 3′, respectively. PCR conditions were as follows: initial denaturation (2 min at 94°C), 35 cycles of denaturation (30 s at 94°C), annealing (30 s at 45°C) and elongation (2 min at 68°C) for *Tb*AS-A, and annealing (30 s at 50°C) and elongation (2 min at 68°C) for *Tc*AS-A, and a final extension step (10 min at 68°C). The PCR products were isolated from a 1% agarose gel, purified by the Qiaex II protocol (Qiagen), and cloned into a pGEM-T Easy vector (Promega) and sent to Eurofins MWG (Germany) for sequencing.

### Expression and purification of poly-His-tagged recombinant *Tb*AS-A and *Tc*AS-A

The *TbASA* and *TcASA* genes were subcloned into pET28a(+) expression vector (Novagen). The recombinant 6-His-tagged proteins were expressed in *E. coli* BL21DE3 by induction of log-phase cultures with 0.5 mM IPTG (NZYTech) for 3 h at 37°C (*Tc*AS-A) and at 18°C, overnight (O/N) (*Tb*AS-A). Bacteria were harvested and resuspended in buffer A [0.5 M NaCl (Sigma), 20 mM Tris.HCl (Sigma), pH 7.6]. The sample was sonicated and centrifuged to obtain the bacterial crude extract. The recombinant proteins were purified using Ni^2+^ resin (ProBond) and washing and elution with increasing levels (25 mM to 1 M) of imidazole (Sigma). The presence and purity of the recombinant protein in the several fractions was determined by SDS-PAGE and Coomassie staining. Dialysis was performed against PBS [137 mM NaCl (Sigma), 2.7 mM KCl (Sigma), 10 mM Na_2_HPO_4_.2H_2_O (Riedel-de Haën), 2 mM KH_2_PO_4_ (Riedel-de Haën) pH 7.4].

To generate rat and rabbit polyclonal antibodies against *Tb*AS-A, each animal was first immunized with 150 µg of recombinant *Tb*AS-A protein. After 2 weeks, 4 boosts with 100 µg of recombinant *Tb*AS-A were given weekly. The collected blood samples were centrifuged to obtain the serum.

### Protein extracts and western blot analysis

Extracts were obtained in RIPA buffer [(20 mM Tris-HCl (Sigma) (pH 7.5), 150 mM NaCl (Sigma), 1 mM Na_2_EDTA (Sigma), 1 mM EGTA (Sigma), 1% Nonidet P-40 (Sigma), 1% sodium deoxycholate (Sigma), 2.5 mM sodium pyrophosphate (Sigma), 1 mM β-glycerophosphate (Sigma), 1 mM Na_3_VO_4_ (Sigma)], with freshly-added complete protease inhibitor cocktail (Roche Applied Science). The total protein amount was quantified using Biorad Commercial Kit (Reagents A, B and S) and the samples were then kept at −80°C. For analysis of parasites from mice, trypanosomes were purified from mouse blood using a DE-52 (Whatman) column [40].

For Western blotting, 2 µg of recombinant *Tb*AS-A and *Tc*AS-A proteins, 10 µg of total soluble cell extract, or 1×10^7^ parasites, were resolved in SDS/PAGE and transferred on to a nitrocellulose Hy-bond ECL membrane (Amersham Biosciences). The membrane was blocked in 5% (w/v) non-fat dried skimmed milk in PBS/0.1% Tween-20 (blocking solution), followed by incubation with an anti-His-tag rabbit antibody (MicroMol-413) (1∶5000) or a combination of an anti-*Tb*AS-A rabbit antibody (1∶1000) with an anti-aldolase rabbit antibody (1∶5000) in blocking solution at 4°C O/N, respectively. Blots were washed with PBS/0.1% Tween-20 (3×15 min). Horseradish peroxidase-conjugated goat anti-rabbit IgG (Amersham) (1∶5000 for 1 h, at room temperature) in blocking buffer was used as the secondary antibody. The membranes were developed using SuperSignal WestPico Chemiluminescent Substrate (Pierce). ImageJ software (version 1.43u) was used for protein bands semi-quantification.

### Enzyme assays

AS activity was assessed by quantification of asparagine formation [41]. The reactions were performed in a total of 150 µl of enzyme assay mixture in 85 mM Tris-HCl (Sigma) containing aspartate (Sigma), ammonia (Sigma), ATP (Sigma) and 8.4 mM Mg^2+^ (Sigma). Following incubation for set times at 37°C, enzymatic reactions were terminated by boiling 4 min, and then centrifuged at maximum speed for 30 s. 100 µl of the reaction mixture supernatant was added to 900 µl of ninhydrin 0.05% in ethanol. The resulting mixtures were boiled at 100°C for 5 min, then centrifuged for 30 s and maintained on ice. 300 µl of clear supernatant fluids were transferred to 96-well plates, and the absorbance at 340 nm determined [41]. Based on reaction linearity studies, 7.5 µg of enzyme and 15 min incubation at 37°C were selected as final conditions. To determine *K*_m_s, the concentrations of substrates were varied in the following ranges: 1.25–20 mM (aspartate), 0.78–50 mM (ammonia) and 0.62–10 mM (ATP), while the remaining substrates concentrations were in excess ([aspartate] >20 mM, [ATP] >10 mM, and [ammonia] >50 mM). *K*_m_ for glutamine was determined using a concentration range of 1.5625–25 mM, while ATP and aspartate were maintained in excess. Measurements were performed in triplicate, and the initial rate was analyzed to obtain values of *V*_max_ and *K*_m_ by curve fitting using GraphPad Prism (5.0 version).

Using a query based on L-cysteine-S-sulfinic acid inhibitor [42], the ZINC database was screened using the program ROCS (version 2.3.1) to find compounds that have good shape similarity (measured by 3D Tanimoto) and similar functional group overlap to the query molecule. L-cysteine-S-sulfate (Sigma; PubChem Substance ID 24892471) was used under the following conditions: 2.5 mM aspartate, 1.25 mM ATP, 12.5 mM ammonia, and 8.4 mM Mg^2+^. The characterization of the mechanism of inhibition consisted in the determination of *K*_m_ and *V*_max_ for each substrate, in the presence of four inhibitor concentrations (0.025, 0.050, 0.1 and 0.2 mM). The following substrate concentration ranges 1.25–10 and 1.25–50 mM were used for aspartate and ammonia, respectively, while to determine *K*_m_ for ATP, a range from 0.625 to 10 mM (*Tb*AS-A) or from 0.3125 to 5 mM (*Tc*AS-A) was assayed. *K_i_* was determined by “*K*_m app_ Method”[43].

### AS-A protein alignments and *Tb*AS-A/*Tc*AS-A homology models

**Figure 1:**
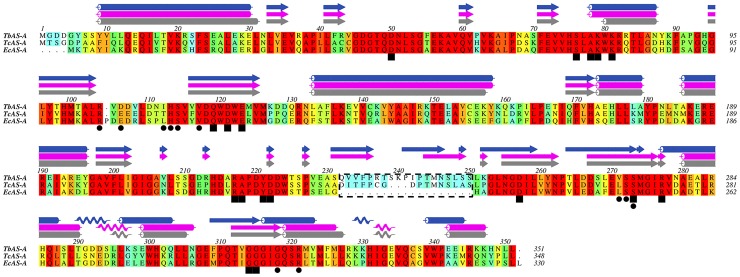
Multiple-sequence alignment of AS-A protein. Alignment of type A asparagine synthetase from *T. brucei* (NCBI-GeneID:3658321/Tb927.7.1110), *T. cruzi* (NCBI-GeneID:3534325/Tc00.1047053503625.10) and *E. coli* (NCBI-GeneID:948258/pdb:12AS). The residues are colored according to ALSCRIPT Calcons (Aline version 011208) using a predefined colour scheme (red: identical residues; orange to blue: scale of conservation of amino acid properties in each alignment column; white: dissimilar residues). Secondary structure elements of *Ec*AS-A crystal structure (grey) and of *Tc*AS-A (purple) and of *Tb*AS-A (blue) homology models are depicted above the alignment. In all protein sequences, asparagine (squares) and AMP binding residues (circles) were identified. The dashed box indicates a structurally divergent region.

*Ec*AS-A, *Tb*AS-A and *Tc*AS-A protein alignments were performed using ClustalW [44]. Aline, Version 011208 [45], was used for editing protein sequence alignments and preparing Fig. 1. *Tb*AS-A and *Tc*AS-A homology models were obtained with SWISS-MODEL, using *Ec*AS-A crystal structure (Protein Data Bank (PDB) accession code 12AS [24]) as a template (percentage of sequence identity of 56% and 57%, respectively) [46]–[48]. The 3D structures were rendered in PyMOL (The PyMOL Molecular Graphics System, Version 1.3, Schrödinger, LLC).

### Generation of *Tb*AS-A RNAi cell lines

The “stuffer strategy” was used to generate RNAi-mediated AS-A depletion. First, the *TbASA* fragment (amplified with a sense oligo with a BglII – SphI linker 5′ - GAGAAGATCTGCATGCGCGACGACGGTTATTCGTCATAC - 3′, and an antisense oligo with a EcoRI – SalI 5′ – CGGAATTCGTCGACACTCCGTTTTTCGGATTGCGGC – 3′) was cloned twice in opposite direction on either sides of a ‘stuffer’ fragment of the pHD1144 vector (also digested with SphI and SalI) (Fig. S1A). The resulting [(target)-stuffer-(reverse-complement target)] construct obtained through HindIII and BglII digestion, which generates a stem-loop RNA, was cloned into pHD1145 (also digested with HindIII and BglII) (Fig. S1B). The final construct was linearized with NotI and 10 µg of DNA was transfected into 2×10^7^/ml bloodstream form cell line carrying pHD1313 plasmid (contains two copies of the tet repressor and a phleomycin resistance cassette) by electroporation using Amaxa Basic Parasite Nucleofector Kit (Lonza). Transcription occurs on induction with tetracycline (100 ng/ml), hence producing mRNA homologs to the target the gene. Stable individual clones were selected 5 to 7 days after transfection with 7.5 µg/ml of hygromycin.

### *In vitro* analysis of *Tb*AS-A RNAi

To analyse growth, *T. brucei* RNAi cell line and cells expressing the tet repressor only (wt), were seeded at 2×10^5^ cells/ml of complete HMI9 medium, in the presence and absence of tetracycline. Cell growth was monitored microscopically on a haemocytometer (Marienfeld) and the culture diluted back to 2×10^5^ cells/ml daily. The same protocol was repeated in complete HMI9 medium with basal IMDM without asparagine, complete HMI9 medium with basal IMDM without asparagine supplemented with 6.7×10^4^ nM of asparagine (levels found in human plasma [49]), and complete HMI9 medium with basal IMDM without asparagine supplemented with 1.67×10^5^ nM of asparagine (levels found in normal medium).

### *In vivo* analysis of *Tb*AS-A RNAi

Wild-type and transgenic bloodstream *T. brucei* parasites were cultured in the absence of selecting drugs (hygromycin and phleomycin) for 24 h, then tetracycline was added. After a further 48 h, parasites were inoculated in mice. For each experiment, 4 groups of BALB/c mice (6–8 weeks old, n = 4) (Harlan Laboratories, United Kingdom) were infected by intraperitoneal injection of 10^4^ *T. brucei* bloodstream forms. 2 groups were injected with wt strain (with or without tetracycline) and the other 2 groups were injected with RNAi cell line (with or without tetracycline). 48 h prior infection the 2 RNAi induced groups were given doxycycline (treated with 1 mg/ml doxycycline hyclate and 5% sucrose containing water). The 2 non-induced groups were given standard water. To evaluate the virulence of RNAi induced parasites in mice with reduced plasmatic levels of asparagine, animals were treated with 50 IU of *E. coli* L- asparaginase (ProSpec-Tany TechnoGene) 48 h before injection and every 48 h. According to the literature, L-asparagine could not be detected in the blood 48 h following an intravenous injection of *E. coli* L-asparaginase, at a dose of 50 IU/mouse [50]. Mice were monitored every day for general appearance and behaviour. Parasitemia was monitored daily from the fifth day post-infection, using tail-vein blood, in a haematocytometer under a microscope. Animals with a parasitemia greater than 10^8^ parasites/ml were euthanized, as previous studies had established that these levels were consistently lethal within the next 24 h.

### Immunofluorescence

*T. brucei* bloodstream forms from log-phase cultures, with or without RNAi, were fixed in μ-Chamber 12 well (Ibidi) for 15 min, at room temperature, in PBS containing 3% p-formaldehyde, washed twice with PBS, and then permeabilized in PBS containing 0.1% of Triton X-100. Fixed cells were incubated for 60 min in PBS containing 10% FCS at room temperature (RT), in a humidified atmosphere, then washed twice with PBS/2% FCS. Cells were then incubated with primary rat or rabbit polyclonal antibody against *Tb*AS-A (1∶100 and 1∶5000 respectively, both diluted in blocking solution) overnight at 4°C, followed by two washes with PBS/2% FCS. Subsequently, cells were incubated with Alexa Fluor 647 conjugated goat anti-rat or Alexa Fluor 488 conjugated goat anti-rabbit secondary antibodies (Molecular probes from Life technologies) (1∶500 diluted in blocking solution) for 1 h at RT in an humidified atmosphere, then washed twice with PBS. Next, the slides were stained and mounted with Vectashield-DAPI (Vector Laboratories, Inc.). Images were captured using fluorescence microscope AxioImager Z1 and software Axiovision 4.7 (Carl Zeiss, Germany). Pseudo-coloring of images was carried out using ImageJ software (version 1.43u).

In case of *Tb*AS-A immunolocalization, *T. brucei* wt bloodstream forms cells were co-stained using rat anti-*Tb*AS-A antibody (1∶100 diluted in blocking solution), rabbit anti-aldolase antibody (1∶5000 diluted in blocking solution), anti-BiP antibody (kindly provided by Dr. Jay Bangs, 1∶500 diluted in blocking solution), anti-enolase antibody (kindly provided by Dr. Paul Michels, 1∶5000 diluted in blocking solution) or anti-GRASP antibody (kindly provided by Dr. Graham Warren, 1∶200 diluted in blocking solution). Alexa Fluor 647 conjugated goat anti-rat (1∶500) and Alexa Fluor 488 conjugated goat anti-rabbit (1∶500) were used as secondary antibodies. Staining with MitoTracker Orange (Invitrogen) followed by Alexa Fluor 488 conjugated goat anti-rabbit (1∶500), as a secondary antibody. The labelling of parasites with MitoTracker was done by adding 250 nM to the cell culture medium (without FCS) for 30 minutes, prior to washing, fixing and staining using the protocol described above. Images were captured using the confocal microscope Leica TCS SP5II and LAS 2.6 software (Leica Microsystems, Germany). Again, image analysis was done using ImageJ version 1.43U software.

### Digitonin permeabilization

For each sample condition, 1.0×10^7^ bloodstream cells were washed once with cold trypanosome homogenization buffer (THB), containing 25 mM Tris (Sigma), 1 mM EDTA (Sigma) and 10% sucrose (Sigma), pH = 7.8. Just before cell lysis, leupeptin (Sigma) (final concentration of 2 µg/ml) and different digitonin (Calbiochem) quantities (final concentrations of 5, 12.5, 25, 50, 100, 150 and 200 ug/ml) were added to 500 µl of cold THB, for cell pellet resuspension. Untreated cells (0 µg/ml of digitonin) and those completely permeabilized (total release, the result of incubation in 0.5% Triton X-100) were used as controls. Each sample was incubated 60 min on ice, and then centrifuged at 2000 rpm, 4°C, for 10 min. Supernatants were transferred to new chilled tubes and 500 µl of cold THB was added to each pellet and then mixed. All fractions were analysed through Western blot as described above.

### Cell cycle analysis

*T. brucei* bloodstream forms were analyzed by flow cytometry for DNA content following RNAi induction. Cells were collected by centrifugation and washed twice with PBS containing 2% FCS. Each 2×10^6^ cells were resuspended in 1 mL of PBS/2% FCS and 3 mL of cold absolute ethanol was added while vortexing. Cells were fixed for 1 hour at 4°C and then washed twice in PBS. 1 mL of staining solution [3.8 mM sodium citrate dehydrate (Sigma), 50 µg/mL propidium iodide (Sigma), 0.5 µg/µL RNAse A (Sigma) in PBS] was added to the cell pellets and vortex. Samples were analysed by FACS (Becton Dickinson) after a incubation at 4°C for 30 min. Data was analyzed by FlowJo software (Ashland, OR).

### Statistical analysis

One-way ANOVA and two-tailed Student's test were used for statistical analysis. Statistical analysis was performed using GraphPad Prism Software (version 5.0), and *p* values≤0.01 were considered to be statistically significant. Asterisks indicate statistically significant differences (*** *p*≤0.001, ** *p*≤0.01).

## Results

### Conservation of AS-A in trypanosomes

One open reading frame that code for a putative AS-A is present in the genomes of *T. cruzi* CL Brener Non-Esmeraldo-like and *T. brucei* TREU927 (http://tritrypdb.org) [29]–[31]. A protein multiple sequence alignment, performed using ClustalW [44], of AS-A from *T. brucei* (Tb927.7.1110, NCBI-GeneID:3658321), *T. cruzi* (Tc00.1047053503625.10, NCBI-GeneID:3534325) and *E. coli* (NCBI-GeneID:948258) is shown in Figure 1. The amino acid residues known to be involved in the active-site formation in *E. coli* [24] are highly conserved within the three sequences (Fig. 1). Protein alignments demonstrated 58% similarity for *Ec*AS-A *versus Tb*AS-A, 60% for *Ec*AS-A *versus Tc*AS-A, and 63% for *Tb*AS-A *versus Tc*AS-A.

**Figure 2:**
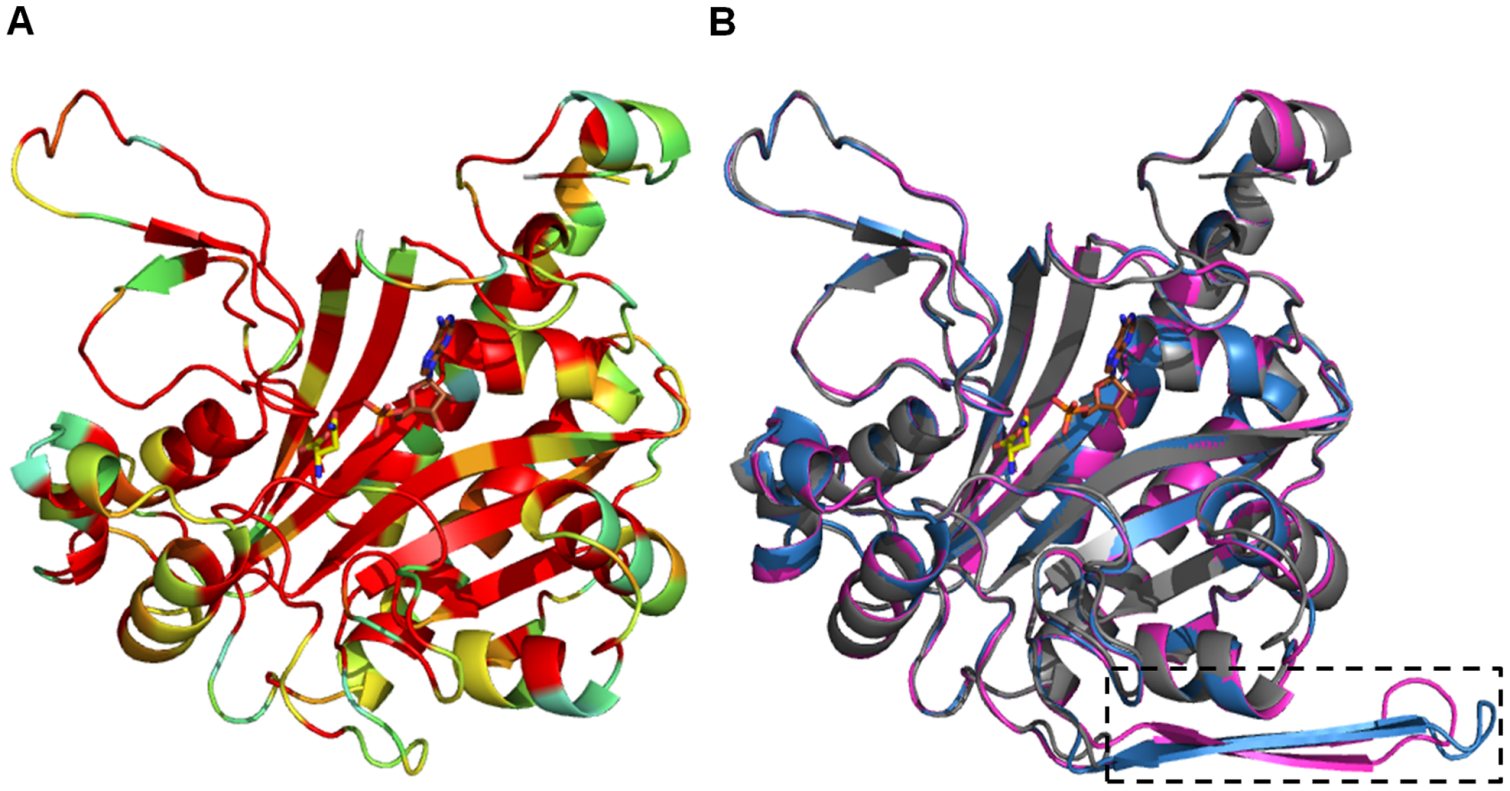
Homology models of AS-A from trypanosomes. (A) Ribbon representation of *Ec*AS-A colored according to the sequence similarity with *Tb*AS-A and *Tc*AS-A as shown in Fig. 1. (B) Superposition of *Ec*AS-A structure (grey) (PDB accession code 12AS), with *Tb*AS-A (blue) and *Tc*AS-A (purple) homology models (obtained from the SWISS-MODEL server, using PDB 12AS as a template). A small structurally divergent region is marked by a dashed rectangle. Ligand color schemes: asparagine is shown in yellow (oxygen, red; nitrogen blue) and AMP is shown in brown (oxygen, red; nitrogen blue; phosphorous orange).

Like *Ec*AS-A, *Tb*AS-A and *Tc*AS are predicted to be dimeric, as seen from superimposed homology models with the *Ec*AS-A crystal structure [24] (Fig. 2A). The only structurally divergent region (area marked by dashed rectangle) (Fig. 1, 2B), is present in both *Tb*AS-A (from residues Q232 to S250) and *Tc*AS-A (from residues D232 to S247), but absent in *Ec*AS-A. This region is distant from the enzyme active site and the dimer interface and its functional and structural significance are unknown. The amino acids involved in asparagine binding are all strictly conserved, while in the AMP binding pocket, the majority of the residues are conserved, except for three residues (Fig. 1). In *Ec*AS-A, E103 (D106 and E106 in *Tb*AS-A and *Tc*AS-A, respectively) and L109 (I112 and T112 in *Tb*AS-A and *Tc*AS-A, respectively) (Fig. 1) are not directly involved in polar interactions with the nucleotide base, but form part of the outer wall of the binding site [24]. The main chain of L249 in *Ec*AS-A (V271 and L268 in *Tb*AS-A and *Tc*AS-A, respectively) is directly involved in hydrogen bonds with ribose from AMP, however the different side chains of leucine and valine do not affect the shape of AMP binding site.

### Trypanosome AS-A catalyze asparagine synthesis using either ammonia or glutamine as nitrogen donors

**Figure 3:**
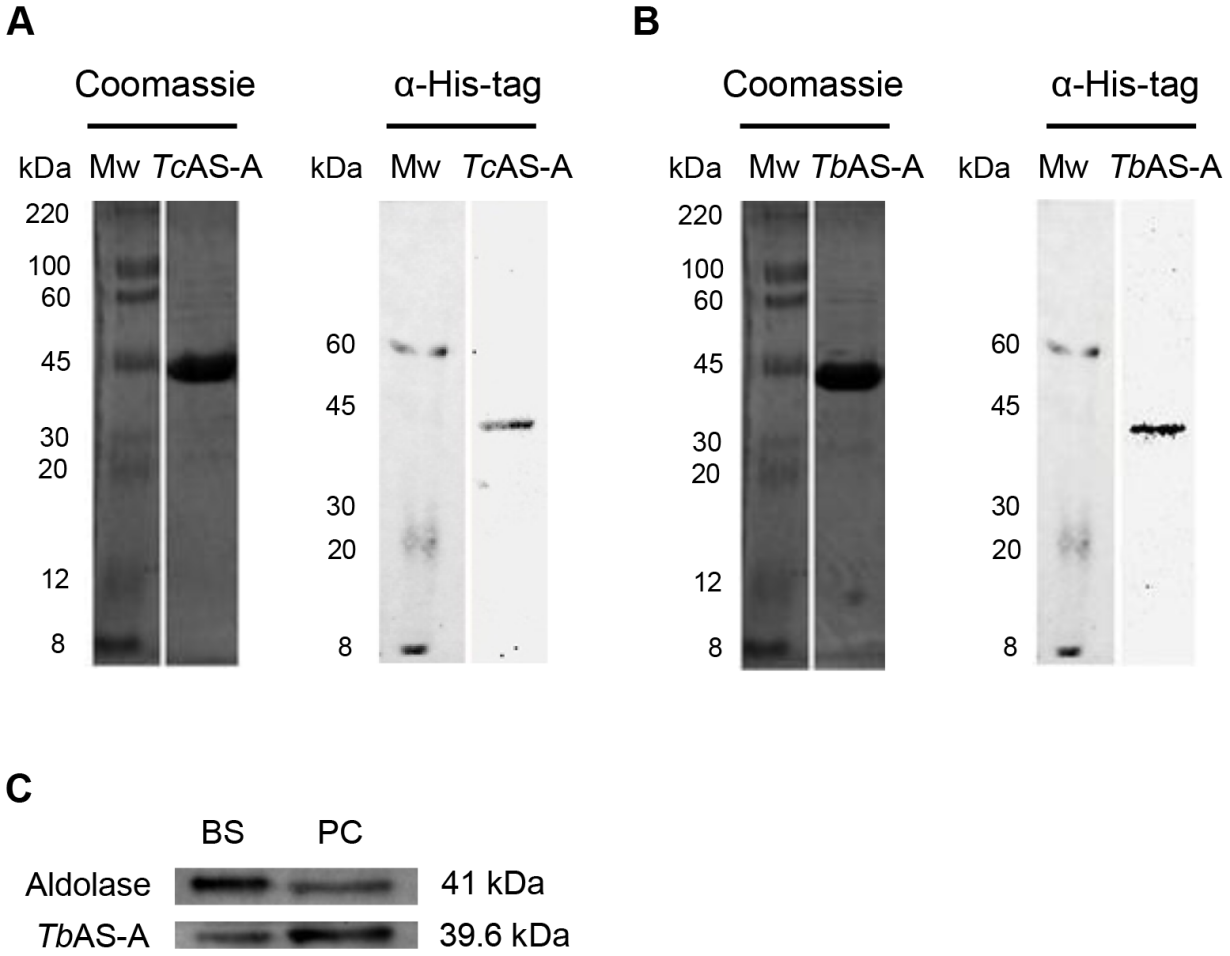
Analysis of purified recombinant *Tc*AS-A/*Tb*AS-A and of AS-A expression within *T. brucei* life cycle stages. Purified *Tc*AS-A (A) and *Tb*AS-A (B) recombinant proteins were analyzed using 12% SDS-PAGE and visualized using Coomassie blue staining. MW, molecular weight marker. Rabbit anti-histidine monoclonal antibody (1∶1000) was used in immunoblotting assays with the *Tc*AS-A (A) and *Tb*AS-A (B) purified recombinant proteins. (C) AS-A expression within *T. brucei* life-cycle stages: 10 µg of protein from BS (bloodstream forms) and PC (procyclic forms) lysates were analysed by Western blot. Aldolase was used as a loading control. Rabbit anti-*Tb*AS-A and anti-aldolase polyclonal antibodies were used for protein detection. The results are representative of three independent experiments.

*Tb*AS-A and *Tc*AS-A coding sequences were cloned into the bacterial expression vector pET28a. Histidine-tagged fusion proteins were purified under non-denaturing conditions (Fig. 3A, B). As expected, the recombinant proteins were recognized by anti-His Tag monoclonal antibody (Fig. 3A, B). Rabbit polyclonal antibodies produced against recombinant *Tb*AS-A recognized the protein in total extracts from two different parasite developmental stages, bloodstream forms (mammalian host parasite stage) and procyclic forms (insect vector parasite stage) (Fig. 3C).

The capacities of *Tb*AS-A and *Tc*AS-A to produce asparagine from aspartate in the presence of ATP, ammonia and Mg^2+^ were determined using a specific quantitative colorimetric assay for L-asparagine [41]. The pH optimum was 7.6, with detectable activity from 6.0 to 9.0 (data not shown). Mg^2+^ was an essential co-factor for *Tb*AS-A and *Tc*AS-A (data not shown), as previously described for *Ec*AS-A [22]. We included 8.4 mM Mg^2+^ in the final reaction mixture. Lower concentrations (2, 4 and 6 mM) gave lower activity while increased concentrations (up to 16 mM) resulted in no substantial activity improvement (data not shown). *Tb*AS-A and *Tc*AS-A showed similar *K_m_*s for aspartate and ammonia (*p*>0.01), while *Tc*AS-A showed higher *K_m_* for ATP than *Tb*AS-A (*p = *0.0042) (Table 1). ATP is the substrate required for the generation of the β-aspartyl adenylate intermediate, which reacts with ammonia, releasing asparagine. In its absence, the reaction did not occur (data not shown). To our surprise, both *Tb*AS-A and *Tc*AS-A could also use glutamine as a nitrogen donor (Table 2). *Tb*AS-A showed higher *K_m_* for this nitrogen donor than *Tc*AS-A, however not statistically significant (*p*>0.01). Both enzymes present higher *K_m_* values for ammonia than for glutamine, but these differences were not statistically significant (*p*>0.01) (Table 1 and 2). *Tb*AS-A had a higher *V*_max_ than *Tc*AS-A, for both ammonia (*p*<0.0001) and glutamine (*p = *0.0043) dependent-activities (Table 1 and 2). *Tb*AS-A had similar catalytic rates for both glutamine and ammonia-dependent activities (*p*>0.01), whereas *Tc*AS-A presented a slightly higher, but not statistically significant, rate for glutamine-dependent activity (*p*>0.01) (Table 1 and 2). The high conservation of the active sites and the small amino acid differences identified in the homology models do not allow an accurate structural interpretation of the small differences observed. Indeed, these might have been due to slight differences in the proportion of protein that was correctly folded.

**Table 1:** *Tb*AS-A and *Tc*AS-A kinetic parameters for aspartate, ATP and ammonia.

Species	Substrate	*K*_m_ (mM)	*V*_max_ ×10^−3^(mM.s^−1^)	*k*_cat_ (s^−1^)	*k*_cat_/*K*_m_ (M^−1^.s^−1^)
*T. brucei*	Aspartate	5.39±0.31	6.72±0.14	5.31±0.11	9.85×10^2^
	ATP	1.80±0.32	7.88±0.48	6.22±0.38	3.46×10^3^
	Ammonia	5.55±0.41	7.21±0.16	5.69±0.13	1.03×10^3^
*T. cruzi*	Aspartate	6.45±2.05	3.09±0.40	2.41±0.32	3.74×10^2^
	ATP	0.72±0.01	4.42±0.02	3.45±0.02	4.79×10^3^
	Ammonia	7.75±1.55	3.14±0.21	2.45±0.17	3.16×10^2^

The values are the means ± standard deviation obtained from 3 independent experiments.

**Table 2:** *Tb*AS-A and *Tc*AS-A kinetic parameters for glutamine.

Species	*K*_m_(mM)	*V*_max_ ×10^−3^ (mM.s^−1^)	*k*_cat_(s^−1^)	*k*_cat_/*K*_m_ (M^−1^.s^−1^)
*T. brucei*	8.20±1.70	7.06±0.61	5.58±0.48	6.80×10^2^
*T. cruzi*	15.33±3.66	4.33±0.53	3.38±0.41	2.20×10^2^

The values are the means ± standard deviation obtained from 3 independent experiments.

**Figure 4:**
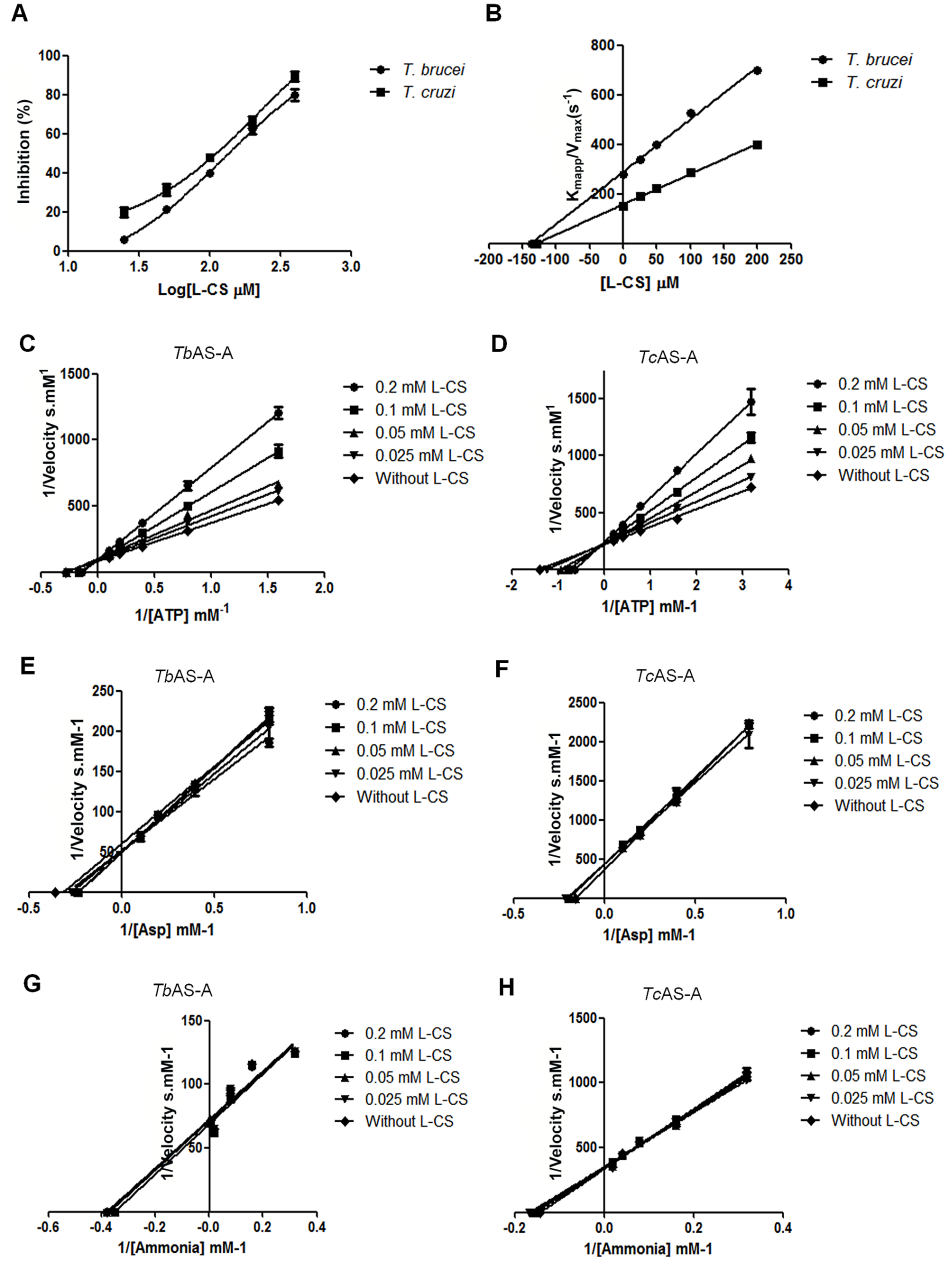
*T. brucei* and *T. cruzi* AS-A *in vitro* inhibition. (A) Inhibition (%) of *T. brucei* and *T.cruzi* AS-A activity by L-cysteine-S-sulfate (L-CS). (B) Plot of *K*_mapp_/*V*_max_ *versus* L-CS concentration was established; *K*_i_ corresponds to the symmetric value of the X-axis intersection. (C-H) Plots showing the effect of different L-CS concentrations on the inverse of the initial velocity *versus* the inverse of several concentrations of ATP, aspartate or ammonia for *Tb*AS-A (C, E and G, respectively) and *Tc*AS-A (D, F and H, respectively) enzymes. Error bars indicate standard deviation of the means of two replicates and data shown are representative of three independent experiments.

L-cysteine-S-sulfate, considered a putative AS-A inhibitor from a virtual screening, inhibited both enzymes, with IC_50_s of 126 and 100 µM for *Tb*AS-A and *Tc*AS-A, respectively (Fig. 4A). For both enzymes, the kinetic characteristics suggested competition with ATP binding (Fig. 4C, D). No changes in the *K*_m_s and *V*_max_s for aspartate and ammonia were observed (*p*>0.01) (Fig. 4E, F, G, H), suggesting the inhibition is exclusively due to ATP binding interference (*p*≤0.01) . *K*_i_ values of 137 and 128.9 µM were determined for *Tb*AS-A and *Tc*AS-A, respectively (Fig. 4B).

### AS-A localizes in the cytosol of *T. brucei* bloodstream forms

**Figure 5:**
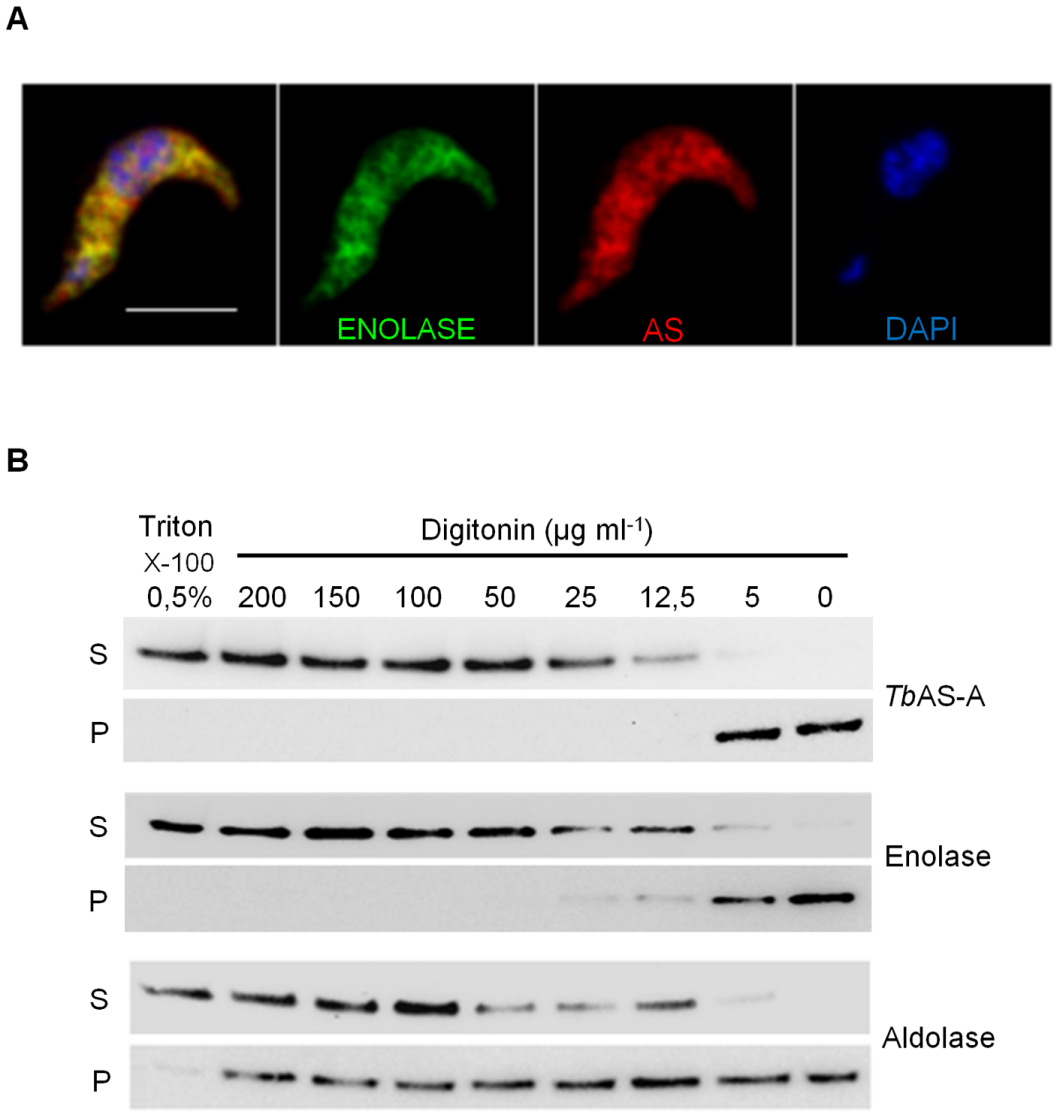
AS-A subcellular localization in *T. brucei* bloodstream forms. (A) Immunofluorescence analysis by confocal microscopy of *Tb*AS-A (red) and enolase (green) in bloodstream forms. DAPI locate nuclear and kinetoplast mitochondrial DNA (blue). Bar, 5 µm. Images are maximal Z-projections of 50 contiguous stacks separated by 0.1 µm. (B) For digitonin permeabilization, selected supernatant (S) and pellet (P) fractions obtained at different digitonin concentrations were subjected to Western-blot analysis and probed with rabbit antisera raised against *Tb*AS-A, enolase (cytoplasmic marker) and aldolase (glycosome marker). Data shown is representative of two independent experiments. Untreated cells (0 µg/ml of digitonin) and those completely permeabilized in Triton X-100 0.5% were used as controls.

The subcellular localization of *Tb*AS-A was determined by immunofluorescence and digitonin fractionation in bloodstream forms. As expected, induction of RNAi resulted in a decrease in the fluorescence intensity (Fig. S2A, B, C). *Tb*AS-A is in the cytosol, as revealed by colocalization with the cytosolic enzyme enolase [51] (Fig. 5A) and no colocalization with aldolase, BiP, GRASP or mitotracker (Fig. S3), markers for glycosomes [52], endoplasmic reticulum [53], Golgi and mitochondria compartments [54], respectively. Controls performed with rat or rabbit pre-immune sera and secondary antibody alone, showed no detectable signal (data not shown). Digitonin fractionation also resulted in similar profiles for AS-A and enolase (cytosolic marker) and no similarity to aldolase (glycosomes marker) (Fig. 5B).

### AS-A knockdown makes *T. brucei* bloodstream forms dependent on extracellular asparagine

**Figure 6:**
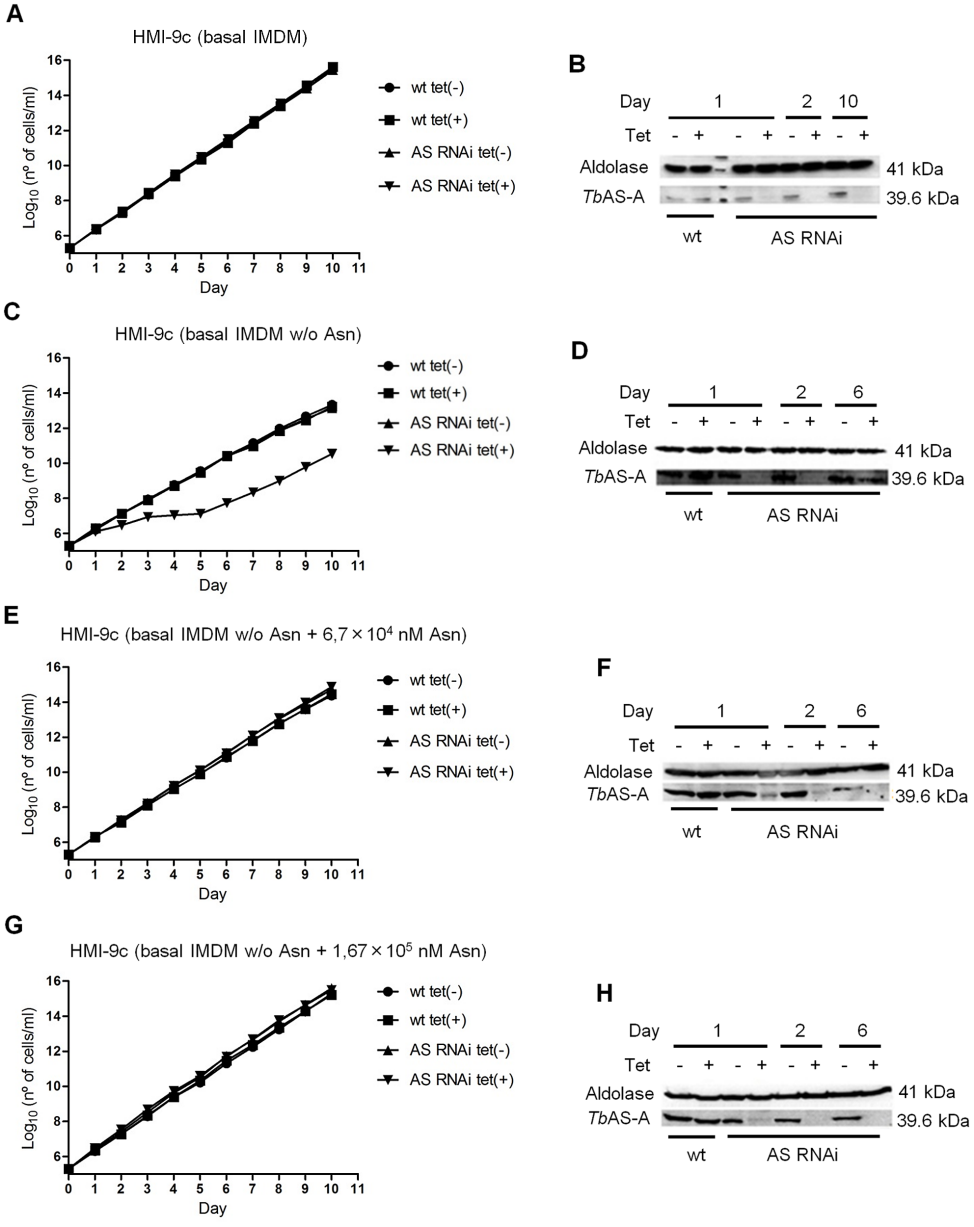
*In vitro* effect of RNAi-mediated AS-A down-regulation in *T. brucei* bloodstream forms. Growth curve of a wt *versus* a representative AS RNAi cell line were performed in unmodified medium [complete HMI-9 (HMI-9c) with basal IMDM] (A), modified medium [HMI-9c with basal medium without asparagine - HMI-9c (basal IMDM w/o Asn)] (C), modified medium supplemented with 6.7×10^4^ nM of asparagine [HMI-9c (basal IMDM w/o Asn + 6.7×10^4^ nM Asn)] (E), modified medium supplemented with 1.67×10^5^ nM of asparagine [HMI-9c (basal IMDM w/o Asn + 1.67×10^5^ nM Asn)] (G). Circles and squares represent wild-type growth in the absence or presence of tetracycline, respectively, while up triangles and down triangles represent clones growth in the absence or presence of tetracycline, respectively. Cumulative cell numbers are plotted as the product of cell number and total dilution. Error bars indicate standard deviation. The effect of RNAi on the AS-A protein levels was analyzed by Western blots with extracts of noninduced tet(−), and RNAi-induced tet(+) cells, isolated from unmodified medium (B), modified medium (complete HMI-9 medium with basal IMDM without asparagine) (D), modified medium supplemented with 6.7×10^4^ nM of asparagine (F), modified medium supplemented with 1.67×10^5^ nM of asparagine (H). Cells were collected at day 1, 2, 6 and 10 of RNAi induction. Results are representative of three independent experiments.

**Figure 7:**
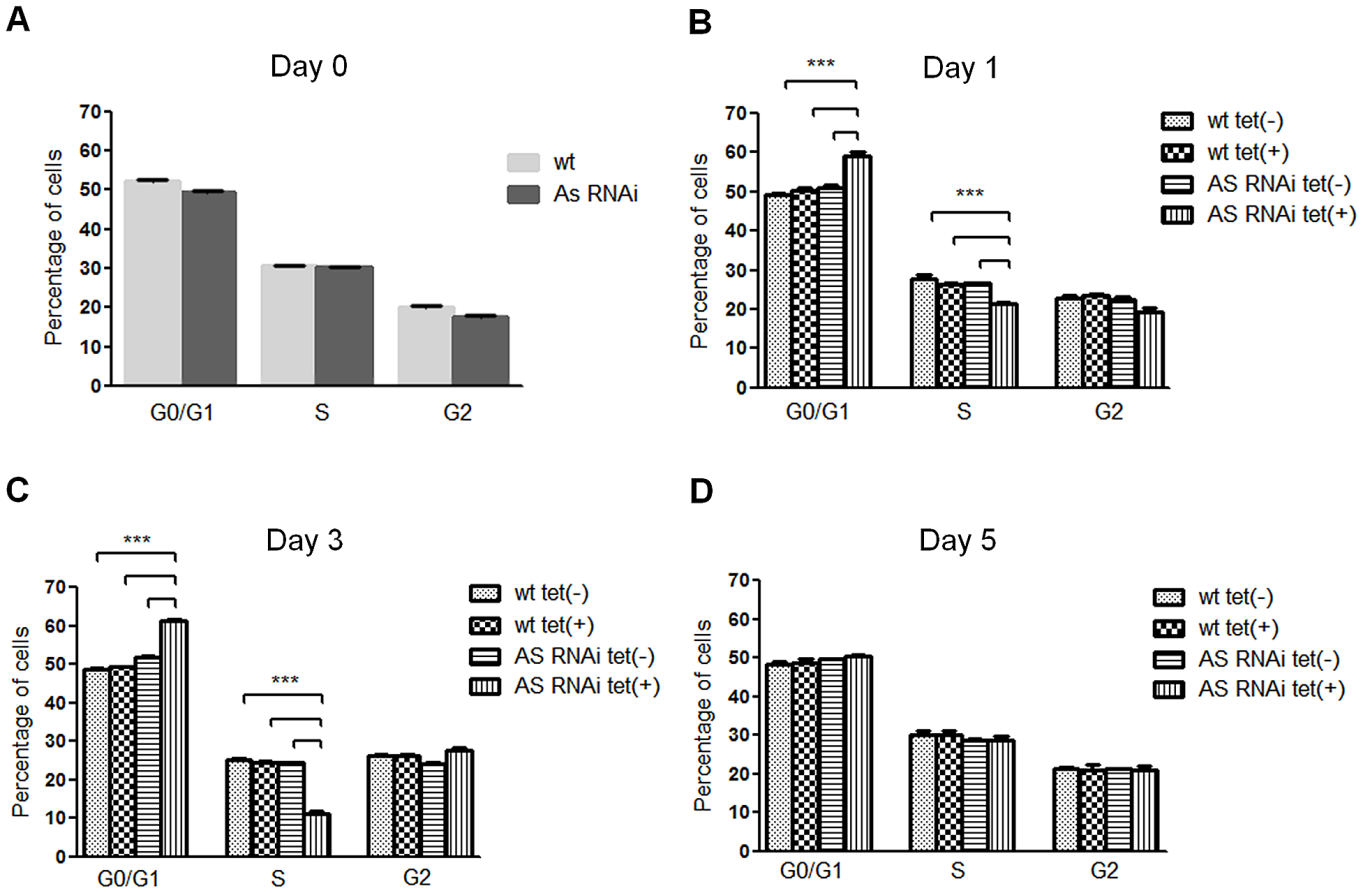
*In vitro* effect of RNAi-mediated AS downregulation on *T. brucei* cell cycle. DNA content of ≈2×10^6^ wt and RNAi cell line parasites grown in completed HMI-9 medium with basal IMDM without asparagine, was collected before tetracycline induction, day 0 (A), and at day 1 (B), 3 (C), and 5 (D) post-induction, for cell cycle analysis. Samples were analysed by flow cytometry and the percentage of cells in each phase of the cell cycle were determined using FlowJo software. Each bar represents the average from three replicates. Error bars indicate standard deviation of these measurements. The statistics were calculated by one-way ANOVA (*** *p*≤0.001 and ** *p*≤0.01).

To study the biological role of AS-A in *T. brucei* bloodstream forms, cells were stably transfected with an RNA interference plasmid construct. RNAi against asparagine synthetase A was induced in normal medium (complete HMI-9) by adding tetracycline. No difference was observed in cell proliferation between induced and non-induced cells (Fig. 6A), although AS-A protein was reduced to ≈13% of the normal level within 48 hours (Fig. 6B). When, however, the AS-A-depleted cells were grown in HMI-9 medium with only the asparagine from the fetal calf serum, growth was impaired, with an increase in the proportion of cells in G0/G1 (Fig. 6C and 7B, C). Presumably the asparagine from the serum allowed this slower growth. Levels of asparagine usually found in human serum (6.7×10^4^ nM) [49], which are somewhat lower than in normal medium (1.67×10^5^ nM; IMDM - Iscove's modified Dulbecco's basal medium from Gibco Invitrogen), were sufficient to overcome this defect (Fig. 6E, G). In complete HMI-9 medium, with only asparagine from the fetal calf serum, the growth defects of induced RNAi clones are abrogated at day 5 post-induction (Fig. 6C, 7D), and the percentage of cells in GO/G1 and S phases of the cell cycle return to the ones found in non-induced cells (Fig. 7A), suggesting the appearance of RNAi revertants, as is also visible on the Western blot (Fig. 6D). Similar reversion to evade lethal RNAi in trypanosomes has been seen many times before [55]. In the presence of asparagine, low AS-A levels were maintained (Fig. 6B, F and H).

Normal *T. brucei* parasites also showed a statistically significant slower growth under conditions of asparagine limitation (*p*≤0.01; data not shown) (compare Fig. 6C, with A, E, G). It is therefore possible that even when the parasite has AS, it also requires external asparagine for optimal *in vitro* growth.

### AS-A is dispensable for *T. brucei* infectivity in mice

**Figure 8:**
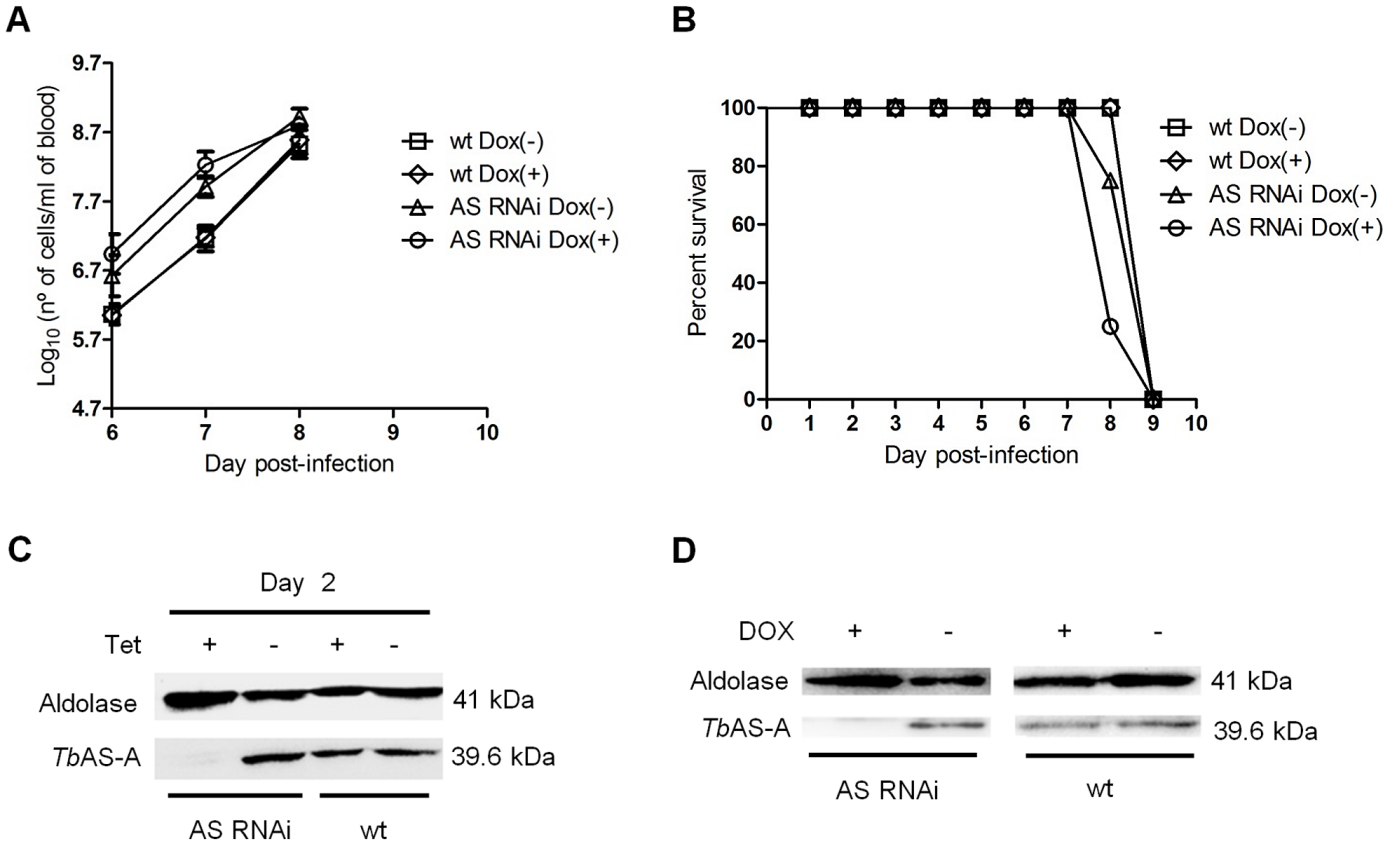
*In vivo* effect of RNAi against *Tb*AS-A. (A) Groups of 4 mice were infected intraperitoneally, with 1×10^4^ control wt parental cell line (open squares and open diamonds) or a representative AS RNAi clone (open triangles and open circles). The mice were either untreated (open squares and open triangles) or treated with 1 mg/ml doxycycline (Dox) (open diamonds and open circles) in the water supply. Parasitemia was quantified at the times indicated. Error bars indicate standard deviation of the means of three mice for wt cell lines, and four mice in the case of AS RNAi cell lines. The detection limit for this assay is 5×10^4^ trypanosomes per ml of blood. Mice were euthanized when parasitemia reached 1×10^8^ cells/ml. (B) Kaplan–Meier survival analysis of mice infected with wt parental cell line and AS RNAi cell line in the absence or presence of doxycycline. Data are representative of three independent experiments. Western blot analysis of the AS-A levels in trypanosomes injected in mice, after 48 h of *in vitro* tetracycline induction (C), and in trypanosomes isolated from mice, before being euthanized (D).

To test whether AS-A is important for parasite infection in a disease model, two groups of mice (*n* = 4) were inoculated with the parental cell line, and other two groups with RNAi cells. Two mice groups were fed with water containing doxycycline to induce down-regulation of *Tb*AS-A, while the remaining mice were kept as non-induced controls. Within six days of inoculation, all mice from the different groups developed high levels of parasitemia (Fig. 8A), and all had to be euthanized after seven or eight days post-infection (Fig. 8B). The results confirm that the asparagine in mouse blood is sufficient to compensate for the ≈87% downregulation of AS-A (Fig. 8C, D).

**Figure 9:**
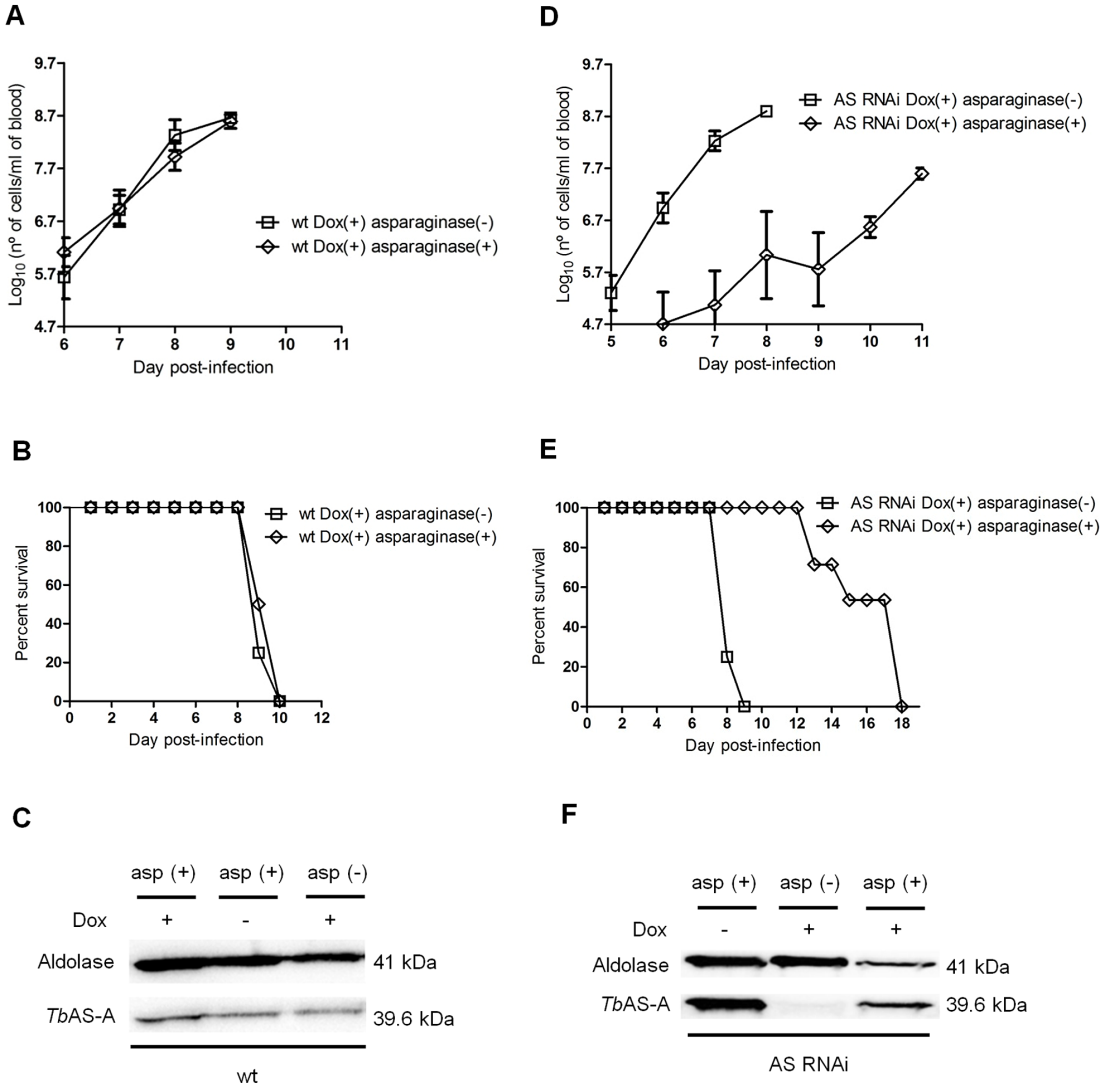
*In vivo* RNAi against *Tb*AS-A in mice undergoing *E. coli* L-asparaginase treatment. Time course of the parasitemia of two groups of 4 mice infected intraperitoneally, with 1×10^4^ of RNAi induced [using 1 mg/ml of doxycycline, Dox (+)] wt parental cell line (A) or a representative AS RNAi cell line (D) either untreated (open squares) or treated daily every 2 days (open diamonds) with 50 IU *E. coli* L-asparaginase/mouse. Parasitemia was quantified in peripheral tail blood at the times indicated. Error bars indicate standard deviation, and data are representative of two independent experiments using two different AS RNAi clones. The detection limit for this assay is 5×10^4^ trypanosomes/ml of blood. Mice were culled when parasitemia reached 1×10^8^ cells/ml. Kaplan–Meier survival plot of mice infected with RNAi induced wt cell line (B) or AS RNAi cell line (E) either untreated (open squares) or treated (open diamonds) with *E. coli* L-asparaginase. (F) Western blot analysis of AS-A levels in trypanosomes isolated from mice, before being euthanized. (C) Wt cell extracts, also collected when mice were culled, were use as negative control.

To assess the contribution of blood L-asparagine *in vivo*, mice were treated with L-asparaginase [50]. L-asparaginase treatment did not affect growth of normal parasites in mice (Fig. 9A) and consequently did not extend animal survival (Fig. 9B). However L-asparaginase treatment in mice infected with *Tb*AS-A RNAi-induced parasites caused a decrease in the parasitemia (Fig. 9D), thus leading to an increase of mice survival (Fig. 9E). Even so, the infection resulted in death. As happened *in vitro*, RNAi revertants appeared during the course of infection in asparaginase-treated, but not untreated, mice (Fig. 9F). Parasites extracts from wt infected mice were used as controls (Fig. 9C).

## Discussion

In this study we demonstrated that trypanosomes AS-A use both ammonia or glutamine as nitrogen donors for the ATP dependent conversion of aspartate into asparagine. Such hybrid activity was only previously demonstrated for type B enzymes, which prefer glutamine to ammonia [15]–[19]. The small differences in *K_m_* of *Tb*AS-A and *Tc*AS-A for ammonia and glutamine (1.5 and 2 fold, respectively) are lower than the difference found in most AS-B enzymes, with the exception of the human enzyme, which has similar affinities for both [16]–[20]. Purified *E. coli* AS-A used only ammonia as the nitrogen source, and results from *Klebsiella aerogenes* also suggested that AS-A preferentially uses ammonia as substrate [5], [21], [22]. The conclusions for AS-A enzymes of these two Gram-negative organisms relied on both biochemical and genetic analysis, but given technical limitations at the time, and the fact that background enzyme activity was seen in the absence of both ammonia and glutamine, some re-examination in bacteria would be worthwhile. Moreover the overall *K_m_* values of trypanosomes AS-A for aspartate, are 6 up to 20 fold higher than the ones found in the literature for prokaryotic asparagine synthetase type A [5], [21], [22]. *Trypanosoma* AS-A structures were not yet been solved and our protein homology models are not completely enlightening, nevertheless we can speculate that such differences may result in the fact that parasite enzymes were expressed and purified as recombinant proteins in bacteria and not purified directly from trypanosomes extracts. As a consequence, differences in protein post-transcriptional processing and/or changes in protein conformation cannot be excluded.

Our results suggest that bloodstream-form parasites rely on two major sources of asparagine to ensure normal proliferation: uptake from the extracellular medium and biosynthesis by AS-A. Bloodstream form proliferation, either *in vitro* or *in vivo*, was only significantly affected when both asparagine sources were compromised. Also consistent with this idea, in the published RNAi screen, a very slight (possibly insignificant) growth disadvantage was seen in bloodstream forms depleted of AS-A [56]. In the same way, our *in vitro* results are corroborated with previous studies, as mammalian cells with low expression of AS are similarly susceptible to asparagine depletion [57]–[59], and asparaginase isolated from *E. coli* and *Erwinia carotovora* act as potent anti-leukemic agents [60].

In the trypanosomes genome there is a second open reading frame (Tb927.3.4060) coding for a putative AS domain. However this is apparently not a classical AS, despite the presence of a good Pfam AS domain (PF0073) at the C-terminus. A BLASTp search using the *T. brucei* sequence revealed a variety of proteins of unknown function that aligned not only across the AS domain, but also in the N-terminal region, which contains N-terminal aminohydrolase domains. Best matches originate from extremely diverse eukaryotes including a plant, an alga, a member of the fungi and an amoeba. BLASTp against the *Saccharomyces cerevisiae* predicted proteome yielded YML096W, and the reciprocal BLASTp on the trypanosome genome indeed gave Tb927.3.4060 as best match. The function of YML096W is not known, and in a trypanosome RNAi screen no growth defect was seen for Tb927.3.4060 [56].

The capacity of trypanosomes to grow using asparagine from the extracellular environment, and the lack of growth defect when the levels of AS-A are reduced, show that only a combination therapy using both a *Tb*AS-A inhibitor and an extracellular asparagine depletor (e.g. L-asparaginase) or an asparagine transport blocker could inhibit parasite growth. This is not appropriate for African sleeping sickness treatment. A combination that absolutely required simultaneous activities of two different drugs would be wide open to resistance development, and drug combination including an intravenously-introduced enzyme is likely to be both too expensive and logistically inappropriate for treatment of African trypanosomiasis. Moreover, L-asparaginase treatment in cancer results in serious adverse events [61]–[63]. We therefore conclude that AS-A is not a good candidate as a sleeping sickness drug target. Its role in *Trypanosoma cruzi*, however, remains to be established.

## Supporting Information

Figure S1 **RNAi vectors used to generate RNAi-mediated** ***Tb*** **AS-A downregulation.** (A) pHD1144 vector for stem-loop cloning (pSP72 vector with a stuffer fragment); (B) pHD1145 inducible polymerase I vector for insertion of ready-made stem-loops (pHD677 vector without a T7 promoter and with an inducible EP1 promoter and hygromycin resistance cassette, insertion into ribosomal spacer).
(TIF)

Figure S2 **Validation of antibodies against** ***Tb*** **AS-A.** Immunofluorescence analysis of *T. brucei* wt or a representative AS RNAi clone grown in the presence or absence of tetracycline. RNAi induced and uninduced cells were grown for 48 h, then fixed and probed with rat polyclonal anti-*Tb*AS-A (A) or rabbit polyclonal anti-*Tb*AS-A (B) antibody and co-stained with DAPI. Bars, 5 µm. Quantification of *Tb*AS-A fluorescence levels in induced cells (AS RNAi tet(+), *n = *30) and uninduced cells (AS RNAi tet(−), *n = *30), using the rat and the rabbit polyclonal anti-*Tb*AS-A antibodies (C). Data representative of two independent experiments using two different clones. ImageJ software (version 1.43u) was used for fluorescence quantification. *p* value was calculated by Student's t test (*** *p*≤0.001 and ** *p*≤0.01).
(TIF)

Figure S3 ***Tb*** **AS-A cellular localization in** ***T. brucei*** **bloodstream forms.** Immunofluorescence analysis by confocal microscopy of *Tb*AS-A (red) in bloodstream forms. Aldolase (glycosome marker), GRASP (golgi marker), BiP (endoplasmic reticulum marker) and MitoTracker (labels mitochondria) are in green. DAPI locate nuclear and kinetoplast mitochondrial DNA (blue). Bars, 5 µm. Images are maximal Z-projections of 50 contiguous stacks separated by 0.1 µm.
(TIF)
