# Correction: Knockdown of Asparagine Synthetase A Renders *Trypanosoma brucei* Auxotrophic to Asparagine

**DOI:** 10.1371/annotation/eb4faa32-fc8d-43ae-ba92-f34c0c4e5052

**Published:** 2013-12-06

**Authors:** Inês Loureiro, Joana Faria, Christine Clayton, Sandra Macedo Ribeiro, Nilanjan Roy, Nuno Santarém, Joana Tavares, Anabela Cordeiro-da-Silva

The following information was missing from the Funding section:

This work was funded by FEDER funds through the Operational Competitiveness Program – COMPETE and by National Funds through FCT – Fundação para a Ciência e a Tecnologia under the project PEst-C/SAU/LA0002/2011. IL, JF and NS were supported by fellowships from FCT reference SFRH/BD/64528/2009, SFRH/BD/79712/2011 and SFRH/BPD/BIA-MIC/118644/2010, respectively. JT is an Investigator FCT funded by National funds through FCT and co-funded through European Social Fund within the Human Potential Operating Programme. The research leading to these results has also received funding from the European Community’s Seventh Framework Programme under grant agreement No.602773 (Project KINDRED). The COST Action CM0801 ‘New drugs for neglected diseases’ and TRICONT project under ERA-NET New INDIGO have also contributed for this work. The funders had no role in study design, data collection and analysis, decision to publish, or preparation of the manuscript.

