# Supplementary figures and images for: Knockdown of Asparagine Synthetase A Renders Trypanosoma brucei Auxotrophic to Asparagine
Source: PLoS Negl Trop Dis. 2013 Dec 5;7(12):e2578. doi: 10.1371/journal.pntd.0002578 (PMC3854871; doi:10.1371/journal.pntd.0002578)

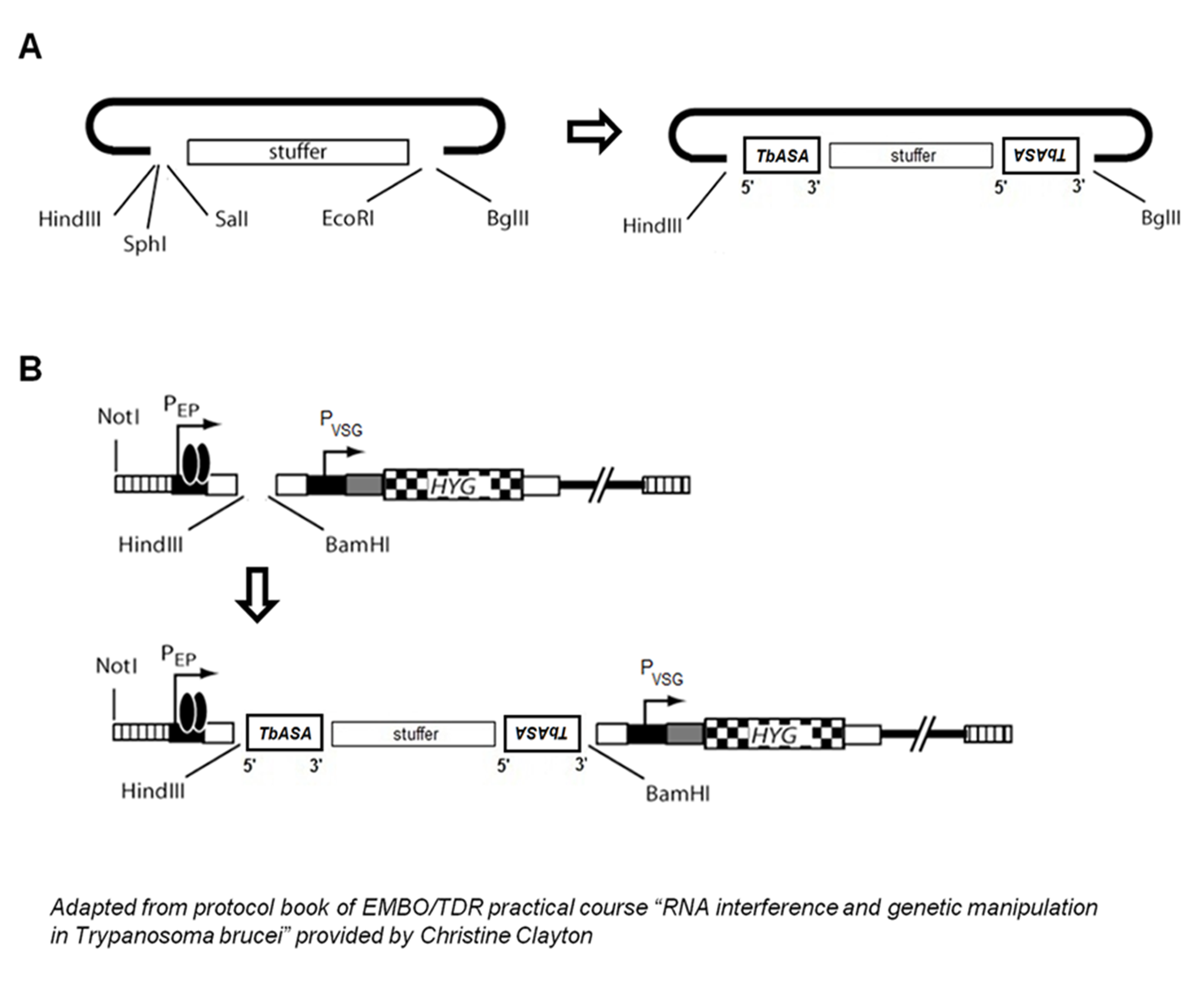

Supplement: Figure S1 — RNAi vectors used to generate RNAi-mediated Tb AS-A downregulation. (A) pHD1144 vector for stem-loop cloning (pSP72 vector with a stuffer fragment); (B) pHD1145 inducible polymerase I vector for insertion of ready-made stem-loops (pHD677 vector without a T7 promoter and with an inducible EP1 promoter and hygromycin resistance cassette, insertion into ribosomal spacer). (TIF) [file pntd.0002578.s001.tif]

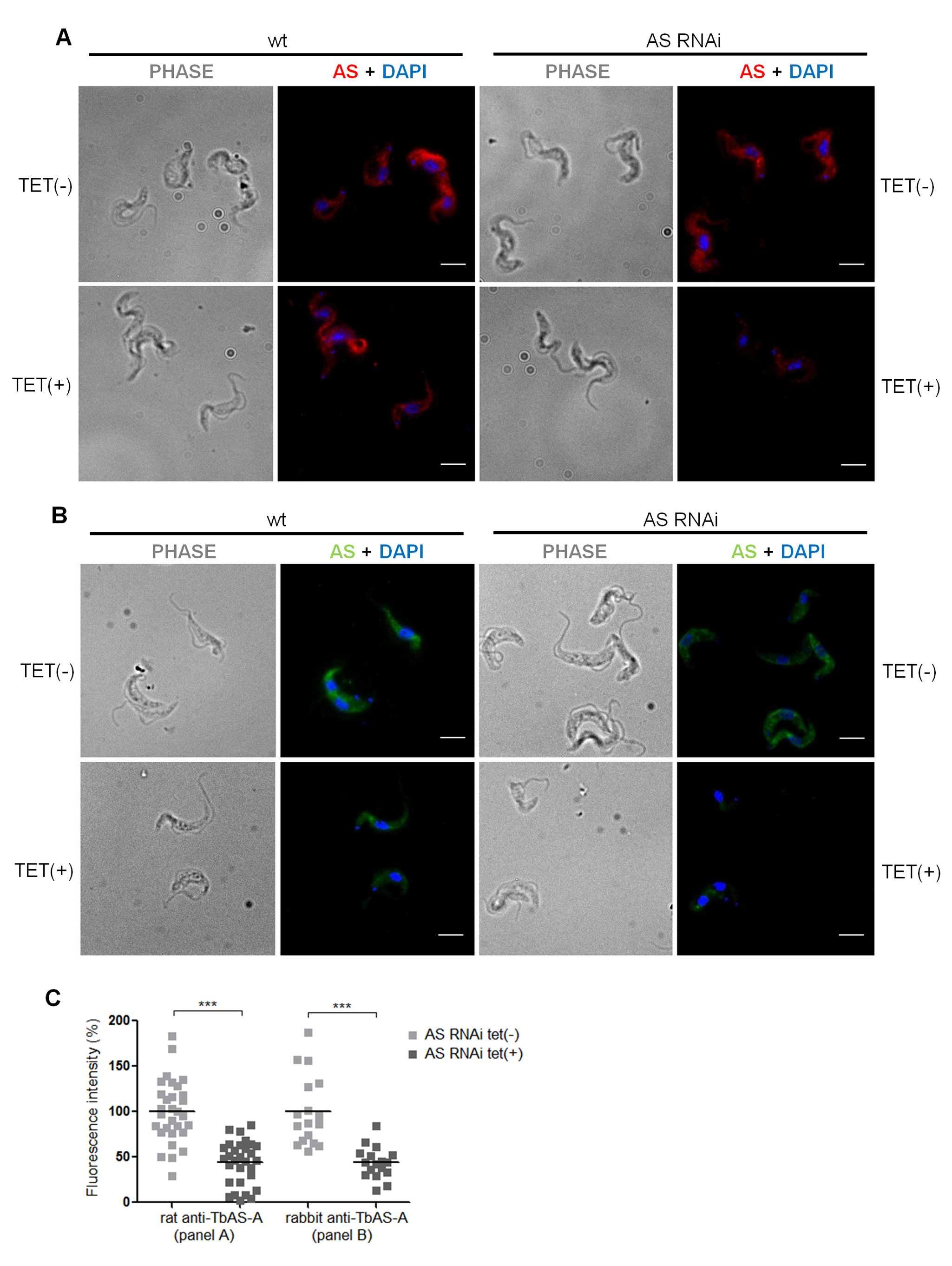

Supplement: Figure S2 — Validation of antibodies against Tb AS-A. Immunofluorescence analysis of T. brucei wt or a representative AS RNAi clone grown in the presence or absence of tetracycline. RNAi induced and uninduced cells were grown for 48 h, then fixed and probed with rat polyclonal anti-TbAS-A (A) or rabbit polyclonal anti-TbAS-A (B) antibody and co-stained with DAPI. Bars, 5 µm. Quantification of TbAS-A fluorescence levels in induced cells (AS RNAi tet(+), n = 30) and uninduced cells (AS RNAi tet(−), n = 30), using the rat and the rabbit polyclonal anti-TbAS-A antibodies (C). Data representative of two independent experiments using two different clones. ImageJ software (version 1.43u) was used for fluorescence quantification. p value was calculated by Student's t test (*** p≤0.001 and ** p≤0.01). (TIF) [file pntd.0002578.s002.tif]

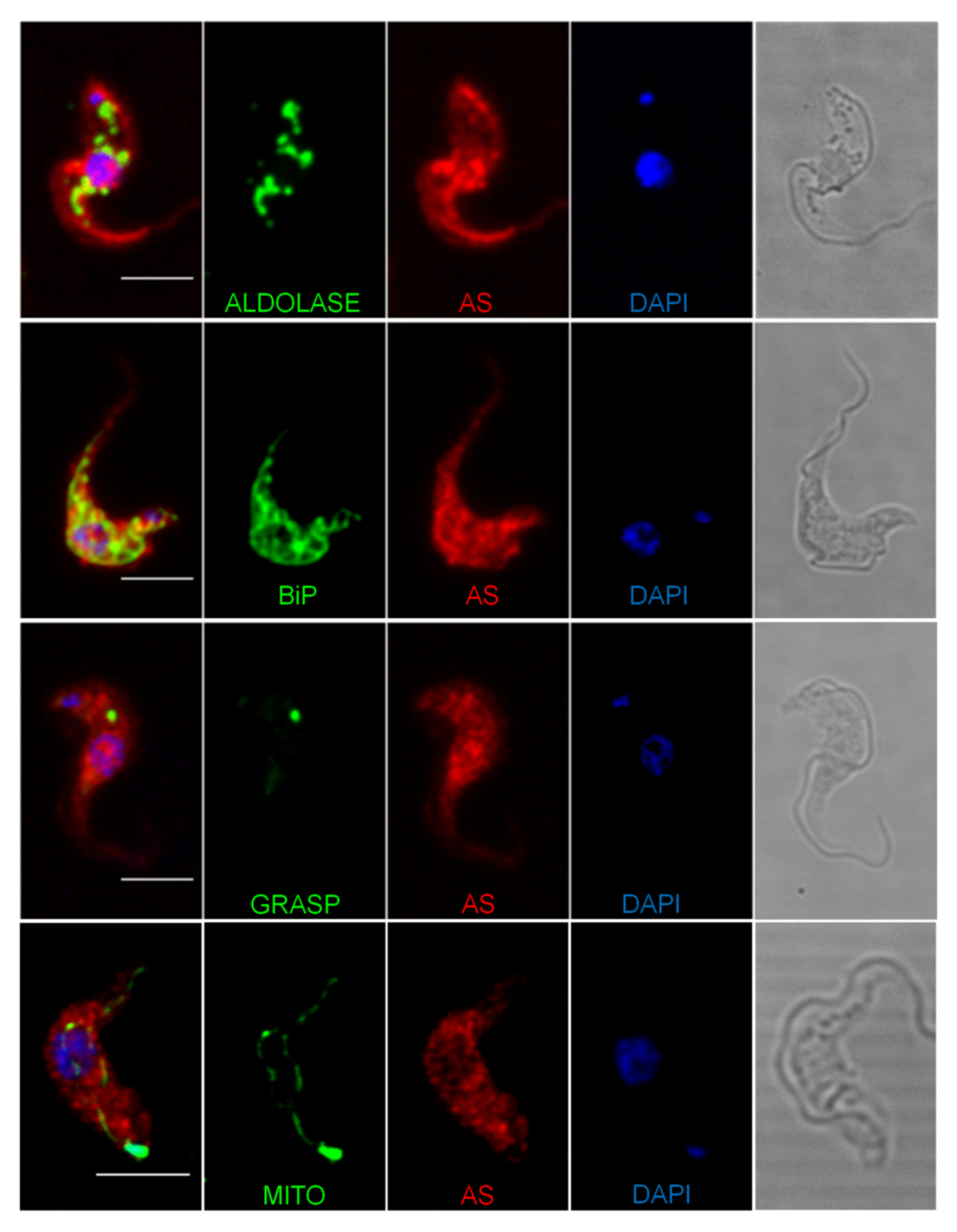

Supplement: Figure S3 — Tb AS-A cellular localization in T. brucei bloodstream forms. Immunofluorescence analysis by confocal microscopy of TbAS-A (red) in bloodstream forms. Aldolase (glycosome marker), GRASP (golgi marker), BiP (endoplasmic reticulum marker) and MitoTracker (labels mitochondria) are in green. DAPI locate nuclear and kinetoplast mitochondrial DNA (blue). Bars, 5 µm. Images are maximal Z-projections of 50 contiguous stacks separated by 0.1 µm. (TIF) [file pntd.0002578.s003.tif]
